# Visual Rhyme Judgment in Adults With Mild-to-Severe Hearing Loss

**DOI:** 10.3389/fpsyg.2019.01149

**Published:** 2019-05-28

**Authors:** Mary Rudner, Henrik Danielsson, Björn Lyxell, Thomas Lunner, Jerker Rönnberg

**Affiliations:** ^1^Linnaeus Centre HEAD, Swedish Institute for Disability Research, Department of Behavioural Sciences and Learning, Linköping University, Linköping, Sweden; ^2^Department of Special Needs Education, University of Oslo, Oslo, Norway; ^3^Eriksholm Research Centre, Oticon A/S, Snekkersten, Denmark

**Keywords:** phonology, frequency, hearing impairment, working memory, lexico-semantic strategy

## Abstract

Adults with poorer peripheral hearing have slower phonological processing speed measured using visual rhyme tasks, and it has been suggested that this is due to fading of phonological representations stored in long-term memory. Representations of both vowels and consonants are likely to be important for determining whether or not two printed words rhyme. However, it is not known whether the relation between phonological processing speed and hearing loss is specific to the lower frequency ranges which characterize vowels or higher frequency ranges that characterize consonants. We tested the visual rhyme ability of 212 adults with hearing loss. As in previous studies, we found that rhyme judgments were slower and less accurate when there was a mismatch between phonological and orthographic information. A substantial portion of the variance in the speed of making correct rhyme judgment decisions was explained by lexical access speed. Reading span, a measure of working memory, explained further variance in match but not mismatch conditions, but no additional variance was explained by auditory variables. This pattern of findings suggests possible reliance on a lexico-semantic word-matching strategy for solving the rhyme judgment task. Future work should investigate the relation between adoption of a lexico-semantic strategy during phonological processing tasks and hearing aid outcome.

## Introduction

Hearing impairment is a highly prevalent neurological condition that is associated with changes in brain organization, i.e., neural plasticity (Jayakody et al., 2018). This applies not only to pre-lingual hearing impairment and profound deafness (Lomber et al., 2010; Rudner, 2018) but also to acquired hearing loss (Ito et al., 1993; Cardin, 2016; Jayakody et al., 2018). Even mild hearing loss leads to neural plasticity (Campbell and Sharma, 2014) and recently we showed hearing-related variation in regional brain volume in a large middle-aged non-clinical cohort (Rudner et al., 2019).

Plasticity associated with hearing loss is found not only in the auditory pathway, including primary auditory cortex (Peelle et al., 2011; Eckert et al., 2012) and auditory processing regions in the superior temporal gyrus (Husain et al., 2011; Boyen et al., 2013; Yang et al., 2014; Rudner et al., 2019), but also in brain regions involved in higher order cognitive processing (Husain et al., 2011; Yang et al., 2014; Rudner et al., 2019), including phonological processing (Lazard et al., 2010; Classon et al., 2013b; Rudner et al., 2013; Andin et al., 2018). This may reflect investment of cognitive resources in listening, especially under adverse conditions (Peelle and Wingfield, 2016; Rudner et al., 2019). Indeed, differences in functional hearing even in a non-clinical cohort are associated with differences in the volume of cognitive processing regions, including the hippocampus (Rudner et al., 2019), a subcortical structure that mediates long-term memory encoding and retrieval.

Long-term memory underpins language processing. To achieve understanding of a spoken message, the incoming speech signal has to be mapped onto knowledge representations stored in long-term memory (Rönnberg et al., 2013, 2019). Phonological representations of the sounds of words, which are dissociable from their lexical representations (Cutler, 2008), are fundamental to this process (Marslen-Wilson, 1987; Luce and Pisoni, 1998), and their importance increases when contextual or lexical information is impoverished (Mattys et al., 2005; Signoret and Rudner, 2019). When conditions for speech perception are optimal (Mattys et al., 2012), the process of matching the speech signal to phonological representations stored in long-term memory is seamless. However, when speech perception conditions are suboptimal, this automatic process may become conscious and effortful, as well as less sensitive to phonological detail (Mattys et al., 2014), and demand the engagement of working memory (Rönnberg et al., 2013, 2019). Although speech perception is relatively robust to minor matching failures, especially when rich context guides lexical selection (Mattys et al., 2012), major mismatch may disrupt processing of the speech signal. The role of working memory in speech understanding under adverse listening conditions includes the piecing together of fragments of target speech along with the suppression of unwanted sounds such as competing speech (Rönnberg et al., 2013, 2019).

Hearing impairment contributes to adverse listening conditions by making the speech signal weaker and reducing its spectral and temporal fidelity (Pichora-Fuller and Singh, 2006). Although hearing aids can amplify sound, their ability to selectively amplify target speech and suppress unwanted sounds is limited (Lunner et al., 2009). There is some evidence that hearing aids may reduce listening effort after a period of familiarization (Rudner et al., 2009; Giroud et al., 2017), improve working memory (Karawani et al., 2018) and slow cognitive decline in later life (Maharani et al., 2018). Hearing impairment may keep working memory in trim by demanding cognitive engagement during speech communication. Long-term memory, however, including the mental lexicon with its set of phonological representations, may decline through disuse (Rönnberg et al., 2011; Classon et al., 2013a).

Considering the key role of phonology in speech understanding, it is not surprising that hearing impairment is associated with changes in phonological processing ability. This applies both to individuals with severe hearing impairment (Andersson, 2002; Lazard et al., 2010; Classon et al., 2013a,c; Lazard and Giraud, 2017). and to individuals with undiagnosed hearing loss (Molander et al., 2013). Andersson (2002) suggested that post-lingual hearing loss leads to degradation of the phonological representations established through use of oral language, and that the mechanism behind this deterioration is the fading of phonological representations that are no longer regularly refreshed by optimal auditory perception.

Phonological processing ability is typically measured using a rhyme judgment task in which participants are asked to compare the sound of different words (Andersson, 2002; Lazard et al., 2010; Classon et al., 2013c). Visual administration circumvents the issue of differences in hearing ability for individuals with hearing impairment. Further, because the sound of words is not directly available in the written word, the participant must actively retrieve phonological representations from long-term memory to make the necessary comparison. This requirement is most obvious in languages that lack a one-to-one mapping between orthography and phonology. For example, in English the non-rhyming words “mint” and “pint” are orthographically similar and thus look as though they might rhyme in the same way as do “mint” and “lint.” In order to determine that “mint” and “pint” do not actually rhyme, the sounds of those words have to be activated and compared. Conversely, “bough” and “cow” do rhyme despite orthographic indication to the contrary. Swedish, the language used in the current investigation, has a more regular mapping of orthography to phonology than English, but there are still examples of mismatch that can be exploited in a visual rhyme task (Andersson, 2002; Classon et al., 2013c). Andersson (2002) argued that the time it takes to determine whether two words rhyme (phonological processing speed) is associated with the quality of phonological representations, such that faster processing indicates better preserved phonological representations. However, Lazard and Giraud (2017) showed that acquired deafness can, in some cases, lead to faster phonological processing, although this phenomenon is associated with maladaptive cross-modal plasticity.

Using a visual rhyme judgment task, Classon et al. (2013c) studied phonological processing in adults with acquired severe hearing impairment. Like Andersson (2002), they found that mean phonological processing speed on trials in which phonology did not match orthography was lower than for adults with normal hearing. However, participants with hearing impairment who had good working memory capacity performed on a par with participants with normal hearing. Classon et al. (2013c) suggested that good working memory capacity facilitates good performance, possibly by allowing repeated double-checking of spelling and sound. Intriguingly, Classon et al. (2013c) also found that those same individuals had poorer subsequent recognition of items from mismatch conditions than their hearing-impaired peers with poorer working memory. Therefore, they suggested that when explicit processing resources are engaged during the overtly phonological strategy of repeated double-checking of spelling and sound, they become unavailable for deeper processing of the lexicosemantic characteristics of the stimuli which is required for long-term memory encoding (Craik and Tulving, 1975). A phonological strategy, involving grapheme to phoneme matching and related to good working memory capacity (Classon et al., 2013c), seems to be more predictive of successful auditory rehabilitation in persons with severe hearing impairment than a lexico-semantic strategy, involving whole-word matching (Lazard et al., 2010; Classon et al., 2013c) and it has been suggested that it may be appropriate to include phonological training in hearing rehabilitation (Moberly et al., 2017).

Rhyme judgment requires the activation of phonological representations relating to the final syllables of the relevant words. These representations reflect the sublexical patterning of phonemes including consonants and vowels. A double dissociation of the processing of consonants and vowels in neuropsychological patients shows that these are distinct categories of speech sounds (Caramazza et al., 2000). Vowels seem to provide more information than consonants when it comes to word segmentation (Mehler et al., 2006) while consonants seem to be more important than vowels for lexical access (Owren and Cardillo, 2006). Both functions are likely to be important for visual rhyme judgment. Vowels are distinguished by their relative formant frequency patterns that occupy a relatively low frequency range while consonants are generally distinguished by their higher frequency characteristics, although there is a large overlap in frequency (Ladefoged, 1993). Thus, based on the notion that hearing loss leads to degradation of phonological representations (Andersson, 2002), hearing loss in the low frequency range is likely to degrade phonological representations of vowel sounds and affect vowel processing while high frequency hearing loss is likely to degrade phonological representations of consonant sounds and affect consonant processing. Indeed, hearing loss seems to have a differential impact on the use of vowel and consonant cues during processing of auditorily gated speech (Fogerty et al., 2012). Further, persons with bilateral, symmetrical, mild-to-severe sensorineural hearing impairment belonging to the same cohort as the participants in the present study showed differences in consonant and vowel processing (Moradi et al., 2017).

The purpose of the present study is to investigate the relationship between phonological processing skills and low and high frequency hearing loss in adults with mild-to-severe hearing impairment. We predict that better phonological processing ability will be associated with better thresholds in both low and high frequency ranges reflecting the relative preservation of both vowel and consonant representations. Different impact of consonant and vowel cues during phonological processing will be indicated by a differential contribution of low and high frequency ranges. We also predict that speed of access to representations in long-term memory will account for additional variance in phonological processing skills along with working memory capacity, especially under mismatch conditions.

## Materials and Methods

The data for the current study were obtained from the N200 database (Rönnberg et al., 2016) under a data transfer agreement. The study was approved by the Regional Ethics Review Board (reference no. 55-09 T122-09) and all participants gave their informed consent.

### Participants

212 individuals (91 female) with a mean age of 60.7 years (*SD* = 8.6 years) were included in the present study. They were all experienced hearing aids users with bilateral, symmetrical sensorineural hearing loss in the range mild-to-severe and were recruited to the longitudinal N200 study from the patient population at the Linköping University Hospital. They all had normal or corrected-to-normal vision and were native Swedish speakers. Data for all participants who had completed the rhyme judgment task at the first time point of the longitudinal N200 study are included in the present study.

### Audiometric Tests

Pure tone audiometry (air-conduction thresholds) was performed in accordance with ISO/IEC 8253 (2010) on all participants in both ears at the frequencies 250, 500, 1000, 2000, 3000, 4000, 6000, and 8000 Hz. We were interested in the contribution of hearing acuity in the vowel and consonant frequency ranges. Vowels are identified most accurately by their first two formant frequencies, with F2 being the more important. Back vowels are likely to have second formant frequencies below 1,000 Hz while front vowels have second formant frequencies above 1,000 Hz, with some above 2,000 Hz. Swedish also has some rounded vowels which rely on third formant frequencies above 2,000 Hz. This means that there is an overlap between the lower frequencies that distinguish vowels and the higher frequencies in the range of 2,000 to 4,000 Hz which distinguish consonants. In order to optimize separation of frequencies distinguishing vowels and consonants while keeping constant the number of data points used, we calculated the better ear pure tone average (PTA) dB across 250, 500 and 1000 Hz for vowel frequency (*M* = 25.31, *SD* = 13.36) and the better ear PTA dB across 2000, 3000, and 4000 Hz for consonant frequency (*M* = 47.95, *SD* = 12.64). We acknowledge that the chosen vowel frequencies do not include the higher formant frequencies for front or rounded vowels. As the frequencies 6000 and 8000 may also contribute to the ability to distinguish consonants, we calculated a third better ear PTA dB across these two high frequencies (*M* = 58.12, *SD* = 16.75). Mean better ear PTA in the three frequency ranges is reported in Table 1.

**Table 1:** Means, standard deviations, and uncorrected correlations with confidence intervals.

Variable	*M*	*SD*	1	2	3	4	5	6	7	8	9	10
(1) Rhyme judgment Mismatch, ms	1891.36	478.96										
(2) Rhyme judgment Match, ms	1449.71	341.30	0.81^∗∗^									
			[0.75, 0.85]									
(3) Age, yrs	60.68	8.65	0.01	0.07								
			[−0.13, 0.14]	[−0.07, 0.20]								
(4) Reading span	15.94	3.91	−0.21^∗∗^	−0.28^∗∗^	−0.35^∗∗^							
			[−0.33, −0.08]	[−0.40, −0.15]	[−0.47, −0.23]							
(5) Vowel freq. dB	25.31	13.36	0.11	0.10	−0.05	0.10						
			[−0.03, 0.24]	[−0.03, 0.24]	[−0.18, 0.08]	[−0.04, 0.23]						
(6) Consonant freq. dB	47.95	12.64	0.09	0.12	0.23^∗∗^	−0.02	0.29^∗∗^					
			[−0.05, 0.22]	[−0.01, 0.25]	[0.10, 0.36]	[−0.16, 0.11]	[0.16, 0.41]					
(7) High freq. dB	58.12	16.75	0.14	0.17^∗^	0.41^∗∗^	−0.16^∗^	−0.08	0.53^∗∗^				
			[−0.00, 0.27]	[0.04, 0.30]	[0.29, 0.52]	[−0.29, −0.03]	[−0.21, 0.06]	[0.42, 0.62]				
(8) Lexical access speed, ms	977.18	202.24	0.65^∗∗^	0.69^∗∗^	0.07	−0.26^∗∗^	0.14^∗^	0.22^∗∗^	0.18^∗∗^			
			[0.56, 0.72]	[0.61, 0.75]	[−0.06, 0.20]	[−0.38, −0.13]	[0.01, 0.27]	[0.09, 0.35]	[0.05, 0.31]			
(9) Physical matching, ms	976.72	206.46	0.39^∗∗^	0.40^∗∗^	0.37^∗∗^	−0.26^∗∗^	0.15^∗^	0.19^∗∗^	0.22^∗∗^	0.56^∗∗^		
			[0.27, 0.50]	[0.28, 0.51]	[0.25, 0.48]	[−0.39, −0.13]	[0.01, 0.28]	[0.05, 0.31]	[0.09, 0.35]	[0.46, 0.65]		
(10) Hearing problems, yrs	14.71	12.11	0.03	0.07	−0.04	0.03	0.32^∗∗^	0.40^∗∗^	0.20^∗∗^	0.07	0.06	
			[−0.10, 0.17]	[−0.07, 0.20]	[−0.18, 0.09]	[−0.11, 0.17]	[0.19, 0.44]	[0.27, 0.50]	[0.07, 0.33]	[−0.06, 0.21]	[−0.07, 0.20]	
(11) Hearing aid use, yrs	6.66	6.48	0.08	0.04	0.16^∗^	0.11	0.42^∗∗^	0.49^∗∗^	0.19^∗∗^	0.05	0.05	0.54^∗∗^
			[−0.06, 0.22]	[−0.10, 0.18]	[0.02, 0.29]	[−0.03, 0.25]	[0.30, 0.53]	[0.38, 0.59]	[0.05, 0.33]	[−0.09, 0.19]	[−0.09,. 019]	[0.43, 0.63]

*M and SD are used to represent mean and standard deviation, respectively. Values in square brackets indicate the 95% confidence interval for each correlation. The confidence interval is a plausible range of population correlations that could have caused the sample correlation (Cumming, 2014). ^∗^ indicates p < 0.05. ^∗∗^ indicates p < 0.01.*

Participants also reported number of years with hearing problems (*M* = 14.71, *SD* = 12.11) and how many years they had used hearing aids (*M* = 6.66, *SD* = 6.48). See Table 1.

### Cognitive Tests

Participants performed a battery of cognitive tests described in detail in Rönnberg et al. (2016). In the current study we focus on rhyme judgment. However, we also take into account performance on tests of working memory (reading span), cognitive processing speed (physical matching) and lexical access (lexical decision) all of which are described below. These variables are included as the theoretical constructs they tap into may all influence phonological processing ability and thus rhyme judgment performance.

#### Rhyme Judgment

In the rhyme judgment task, two printed words are presented on a computer screen and the participant’s task is to judge whether or not they rhyme with each other. There are equal numbers of rhyming and non-rhyming pairs and within both categories there are equal numbers of orthographically similar and orthographically diverse pairs. This means that there are four different kinds of trials:

(1) R+O+. Words that rhyme and are orthographically similar, e.g., FRITT/VITT (i.e., free/white).
(2) R+O−. Words that rhyme but are not orthographically similar, e.g., KURS/DUSCH (i.e., course/shower in Swedish pronounced [ku∫ /du∫ ]).
(3) R-O+. Words that do not rhyme but are orthographically similar, e.g., TAGG/TUGG (i.e., thorn/chew).
(4) R−O−. Words that neither rhyme nor are orthographically similar, e.g., HÄST/TORN (i.e., horse/tower).

There are eight trials in each category. Button-press responses are given and the dependent variable is the mean response time for correct decisions for each of the four different trial types. Previous work has shown that it is harder to solve mismatching (i.e., R+O− and R-O+) than matching (i.e., R+O+ and R−O−) trials (Andersson, 2002; Classon et al., 2013c) and thus we expected longer response times for mismatch than match trials.

#### Physical Matching

In the physical matching test, two printed letters are presented simultaneously on a computer screen and the participant’s task is to determine whether or not they are identical. Button press responses are given. There are 16 trials equally divided between true and false, and the dependent variable is the mean response time for correct trials. This is a measure of cognitive processing speed.

#### Lexical Decision

In the lexical decision task, strings of three letters (two consonants and one vowel) are presented on a computer screen and the participant’s task is to determine whether or not the string constitutes a Swedish word. The strings are Swedish words, Swedish pseudowords (non-lexicalized but phonologically acceptable) or Swedish non-words (neither lexicalized nor phonologically acceptable). Button press responses are given. There are forty trials evenly distributed between true and false with pseudowords and non-words occurring equally often across false trials. The dependent variable is the mean response time for correct trials. This is a measure of lexical access speed.

#### Reading Span

The reading span test (Daneman and Carpenter, 1980; Baddeley et al., 1985; Rönnberg et al., 1989) is a test of simultaneous working memory maintenance and processing. Three-word sentences are presented word-by-word in blocks of 2–5 sentences and the participant’s task is to first determine whether each sentence makes sense or not as it is presented and then at the end of each block recall either the first or the final word (as cued after the block) of each of the sentences included in the block in order of presentation (Lunner, 2003). The sentences are presented on a computer screen at a rate of one word per 800 msec. Half of the sentences make sense and the other half do not. There are two trials per block length, giving a total of 28 trials. The dependent variable was the total number of words correctly recalled.

### Procedure

The data for the present study were collected at one and the same session lasting between 2 and 3 h. This was the first of three testing sessions, constituting the first time point of the longitudinal N200 study (see Rönnberg et al., 2016 for further details). Tests were administered in a fixed order for all participants. The order of the tests included in the present study was: pure tone audiometry, physical matching, lexical decision, rhyme judgment and reading span. Additional tests, not reported in the present study, were interleaved with these tests (for further details see Rönnberg et al., 2016).

### Data Analysis

All analyses were done using R (Version 3.5.1; R Core Team, 2018). There were missing values for 25 data points. These were mainly due to technical problems with the testing equipment and therefore the missing data was considered to be missing completely at random. Imputation of data was done with multivariate imputation by chained equations using the MICE r package (van Buuren and Groothuis-Oudshoorn, 2011). Following the package recommendations, we obtained five different imputed datasets. The descriptive data presented in the article are based on the original data and the analyses are performed on the imputed data.

First, two repeated measures ANOVAs were performed to establish whether rhyme judgment performance in terms of both accuracy and latency was indeed poorer on mismatch conditions where the information provided by the orthography was misleading as regards the phonological similarity of the presented word pair than on match conditions where orthography agreed with phonology. Because we were interested in phonological processing differences under match and mismatch conditions (Classon et al., 2013c), data were collapsed over the two matching conditions R+O+ (Words that rhyme and are orthographically similar) and R−O− (Words that neither rhyme nor are orthographically similar) and over the two mismatching conditions R+O− (words that rhyme but are not orthographically similar) and R−O+ (words that do not rhyme but are orthographically similar). Response time for match conditions and mismatch conditions was then correlated (Pearson) with age, auditory and cognitive variables. Finally, regression analyses were performed to determine the relative contribution of auditory and cognitive variables to phonological processing ability. The assumptions for multiple regression was assessed with the gvlma r package (Pena and Slate, 2014), and were not met regarding skewness and kurtosis. Therefore, robust regression using the lmrob function in the robustbase r package (Maechler et al., 2018) was used. Variable selection in the regression was done using a stepwise forward method based on *p*-values with a cut-off of 0.1. In regression analysis, there is a growing consensus based on simulation studies (Steyerberg et al., 2001) and reviews (Moons et al., 2015) that a higher cut-off value than the standard 0.05 be used so that potentially important variables are not omitted.

## Results

There were no missing values on the rhyme judgment variables, and so it was not necessary to use the five imputed datasets in this analysis. Means, standard deviations and uncorrected correlations for match and mismatch conditions of the rhyme judgment task and other variables are shown in Table 1.

The ANOVA of rhyme judgment accuracy (proportion correct) scores showed a main effect of condition, *F*(1,209) = 498.53, *MSE* = 41,119.68, *p* < 0.001, η^2^ = 0.221, where accuracy, as expected, was greater under match compared to mismatch conditions (*M*_match_ = 0.98, *M*_mismatch_ = 0.77). As rhyme judgment accuracy approached 100% in the match conditions, meaning that variance was skewed, response time was used as an index of phonological processing ability in the correlation and regression analyses. The response time ANOVA also showed a main effect of condition *F*(1,209) = 236.12, *MSE* = 0.02, *p* < 0.001, η^2^ = 0.332, where responses, again as expected, were faster under match compared to mismatch conditions (*M*_match_ = 1450, *M*_mismatch_ = 1891). Together, these two ANOVAs confirm that the rhyme judgment task was solved more accurately and faster under match compared to mismatch conditions.

### Correlations

Correlations are shown in Table 1. In the rhyme judgment data there was a strong positive correlation between match and mismatch conditions, showing that individuals who were faster on mismatch trials were also faster on match trials. Rhyme judgment performance did not correlate significantly with age but it correlated with the cognitive measures under both mismatch and match conditions. These significant correlations were strong regarding lexical access speed and cognitive processing speed, and moderate regarding reading span. Rhyme judgment performance correlated very weakly with audiometric measures. The only correlation that reached significance was between rhyme judgment under match conditions with high frequency hearing thresholds. Previous studies have examined the relation between rhyme judgment and average hearing thresholds across vowel and consonant ranges. To check that the lack of correlation with hearing thresholds in the present study was not due to separation of vowel and consonant frequencies, we calculated correlations for better ear PTA across the four frequencies 500, 1000, 2000, 4000 Hz. Neither of these correlations reached significance, Mismatch: *r* = 0.12, *p* = 0.09; Match *r* = 0.13, *p* = 0.06. To determine the relative contribution of age, auditory and cognitive variables to variance in rhyme judgment performance under match and mismatch conditions regression analyses were performed.

### Regression

Robust regression analysis with stepwise elimination was performed separately for rhyme judgment performance under match and mismatch conditions. Mean response time was the dependent variable. The variables included in the regression were age; hearing thresholds at vowel, consonant and high frequencies, duration of hearing problems and hearing aid use as well as cognitive variables including reading span, lexical access speed and cognitive processing speed. The same remaining variables were found on all five imputed datasets. Therefore, pooled estimates over all datasets are presented. For the mismatch condition, only lexical access speed was a significant predictor, and that model had an adjusted R^*2*^ of 0.45. For the match condition, lexical access speed and reading span both contributed to the model with adjusted R^*2*^ of 0.55. The final models are presented in Table 2. There was no contribution of any auditory variable in either condition.

**Table 2:** Table of estimates from robust regression for mismatch at the top and match conditions at the bottom.

Condition	Predictor	*b*	*se*	*t*	*P*
Mismatch					
	Intercept	317.112	119.009	2.665	0.008
	Lexical access speed	1.592	0.128	12.477	<0.001
Match					
	Intercept	496.214	103.775	4.782	<0.001
	Lexical access speed	1.070	0.080	13.397	<0.001
	Reading span	−7.823	3.653	−2.141	0.033

*These are the final models after variable selection, pooled over all five imputed data sets.*

## Discussion

In the present study, we tested the phonological processing abilities of 212 individuals with varying degrees of hearing impairment in the range mild-to-severe taking part in the N200 study (Rönnberg et al., 2016). We did this by administering a visual rhyme task, and investigated whether variance in latency could be explained by hearing ability across the frequencies 250, 500, and 1000 Hz or across the frequencies 2000, 3000, and 4000 Hz or both ranges. The main finding in the regression analyses was that a substantial amount of the variance in rhyme judgment performance was explained by lexical access speed but that no additional variance was explained by auditory variables. In match but not mismatch conditions, reading span explained additional variance.

The visual rhyme judgment task used in the present study included four different types of trials that fall into two categories: mismatch (R+O−, R-O+) and match (R+O+, R−O−). There were 16 trials in each category. This is fewer than in some previous studies. For example, the study by Andersson (2002) included 25 trials in each category and the study by Classon et al. (2013c) included 96 trials in each category. Fewer trials means fewer word pairs compared for their phonological characteristics, which in turn means that fewer phonemic contrasts are tested for each individual. Thus, the weakness of the association between hearing impairment and phonological processing in the current study may be partly due to the limited number of trials in the visual rhyme judgment task. This limitation is only partly compensated for by the substantial number of data points generated by the large number of participants. It is probably also due to the frequency ranges chosen to represent consonant and vowel perception. A different choice of frequencies may have shown that the auditory factors did correlate with rhyme judgment.

In line with previous work studying individuals with severe hearing impairment (Andersson, 2002; Classon et al., 2013c), results of the current study showed that performance was statistically significantly poorer on mismatch compared to the match trials. This applied both to the proportion correct and to latency. In match conditions, because the phonology matches the orthography, the correct answer can be obtained simply from the visible orthography relating to the two words presented for comparison without activating their phonology. However, because 50% of the trials are mismatches an orthographic strategy only will lead to poor overall performance. To achieve good performance overall, the phonology of the words must be accessed on the basis of the orthography presumably by individual grapheme/phoneme matching.

In the present study, the associations between phonological processing speed and variables of interest including lexical access speed, hearing thresholds and reading span were tested separately for match and mismatch conditions using Pearson’s correlations. Phonological processing speed was strongly associated with lexical access speed and cognitive processing speed. Both these associations were positive showing that individuals with faster lexical access and cognitive processing speed also have faster phonological processing. Associations between phonological processing speed and hearing thresholds were surprisingly weak, considering our predictions, suggesting that phonological representations do not contribute to performance on the rhyme judgment task above and beyond lexical access speed. This is only likely to be the case if a non-phonological strategy is adopted. There was a moderate negative association with reading span (a measure of working memory capacity) showing that faster phonological processing is associated with greater working memory capacity, in line with our prediction.

Regression analysis resulted in two models explaining variance in phonological processing speed. One model related to mismatch conditions and showed that variance was largely explained by lexical access speed, but that there was no contribution of auditory measures; this was not surprising, considering the pattern of correlations. Importantly, although both lexical access speed and cognitive speed correlated with phonological processing speed, cognitive speed was not retained in the final regression model relating to mismatch. This suggests that lexical access speed specifically, rather than cognitive processing speed in general, is important for phonological processing in the sample studied here, in line with our prediction. Surprisingly, reading span did not contribute above and beyond lexical access speed to the model pertaining to mismatch conditions. The other model related to match conditions and showed that variance was, again, largely explained by lexical access speed, but that reading span also contributed. Classon et al. (2013c) showed that individuals with severe hearing impairment but good working memory capacity could make up for their phonological processing deficit in the mismatch conditions of a visual rhyme task by intensifying their efforts at phonological recoding of orthographic information. Thus, we expected that reading span would contribute specifically to phonological processing under mismatch conditions in the present study. However, we found that working memory capacity indexed by reading span only explained variance under match conditions. This suggests that working memory is being employed to support processing in trials in which the orthography directly indicates whether the words rhyme or not rather than in trials that require repeated checking of sound and spelling (Classon et al., 2013c) and thus explicit engagement of phonological processing skills. Classon et al. (2013c) argued that individuals with severe hearing impairment but poor working memory seemed to adopt a lexico-semantic strategy involving whole-word matching to solve the visual rhyme judgment task (Lazard et al., 2010; Classon et al., 2013c). Such a strategy would be consistent with the results obtained in the present study.

Recently, Lazard and Giraud (2017) reported that adults with severe hearing impairment who adopted a lexico-semantic strategy during visual rhyme judgment responded faster but showed right occipito-temporal reorganization in the brain and poor rehabilitation prognosis with cochlear implants. They suggested that accurate but faster-than-average performance on a visual rhyme task may be a marker of maladaptive plasticity in adults with severe acquired hearing impairment. The brain reorganizes even with mild hearing impairment (Campbell and Sharma, 2014), and it is possible that use of a lexico-semantic strategy during visual rhyme judgment may be a marker of maladaptive plasticity even in early-stage hearing impairment. Indeed, we showed that even in a non-clinical cohort, poorer hearing is associated with smaller brain volume in auditory cortex and regions involved in cognitive processing (Rudner et al., 2019). It is important to establish whether phonological processing abilities are reduced even in early stage hearing impairment as indicated by Molander et al. (2013) and whether this is associated with neural reorganization.

Brain plasticity in connection with hearing impairment is both a threat and an opportunity, and auditory rehabilitation should target neural organization associated with good hearing health (Glick and Sharma, 2017). Activation of left lateralized phonological processing networks during visual rhyme judgment in individuals with severe hearing impairment is associated with successful outcome of cochlear implantation (Lazard and Giraud, 2017). The organization of phonological processing networks in individuals with early stage hearing impairment should be investigated to determine possible brain reorganization and its association with rehabilitation outcomes.

One way of maintaining a phonological strategy in preference to a lexico-semantic strategy during word processing may be targeted phonological training (Moberly et al., 2017). Ferguson and Henshaw (2015) proposed that training programs in which cognitive enhancement is embedded in auditory tasks may benefit the real-world listening abilities of adults with hearing impairment and Nakeva von Mentzer et al. (2013) reported evidence of training effects on phonological processing in children with hearing impairment. Future work should investigate whether training can help maintain left-lateralized phonological processing in adults with hearing impairment.

In conclusion, in a group of 212 adults with hearing impairment, we found evidence that auditory measures tested were only very weakly correlated with performance on a visual rhyme judgment task and once lexical access speed was accounted for, auditory measures did not explain additional variance in visual rhyme judgment performance. We tentatively propose that this finding may be explained by reliance on a lexico-semantic word-matching strategy during visual rhyme judgment, making phonological representations redundant or even a source of confusion. Future work should investigate possible neural plasticity in left-hemisphere phonological processing networks of individuals with early-stage hearing impairment and how it can be shaped to promote good hearing health. It would also be beneficial to retest the original prediction that auditory factors would correlate with better performance in rhyme judgment tasks by using a more appropriate set of frequencies to test for consonant and vowel perception.

## Ethics Statement

This study was carried out in accordance with Swedish legislation. All subjects gave written informed consent in accordance with the Declaration of Helsinki. The protocol was approved by the Regional ethics review board, Linköping, Sweden.

## Author Contributions

MR conceived the article and wrote the first draft. MR, HD, BL, TL, and JR designed the study and contributed to the final version of the article. HD performed the statistical analyses.

## Conflict of Interest Statement

The authors declare that the research was conducted in the absence of any commercial or financial relationships that could be construed as a potential conflict of interest.
